# Fatty acid composition and parasitism of European sardine (*Sardina pilchardus*) and anchovy (*Engraulis encrasicolus*) populations in the northern Catalan Sea in the context of changing environmental conditions

**DOI:** 10.1093/conphys/coaa121

**Published:** 2020-12-29

**Authors:** Sebastian Biton-Porsmoguer, Ricard Bou, Elsa Lloret, Manuel Alcaide, Josep Lloret

**Affiliations:** 1 Institute of Aquatic Ecology, Oceans and Human Health Chair, Faculty of Sciences, University of Girona, C/Maria Aurèlia Capmany 69, E-17003 Girona, Catalonia, Spain; 2 Institut de Recerca i Tecnologia Agroalimentàries, Food Technology, Finca Camps i Armet s/n, E-17121 Monells, Catalonia, Spain

**Keywords:** Fatty acids, parasitism, pelagic environment, sea warming, small pelagic fish

## Abstract

The status of sardine and anchovy populations in the northern Mediterranean Sea has been declining in recent decades. In this study, fatty acids and parasitism at different reproductive and feeding stages in these two species were assessed using specimens caught along the northern Catalan coast, in order to assess the links between lipid dynamics, reproduction and feeding in these two species and to contribute towards an explanation of the potential causes of the current poor situation of the stocks. The results support the use of fatty acid levels as indicators of the body condition of sardine and anchovy at different reproductive and feeding stages, as well as that of the pelagic environmental conditions. In particular, the relatively low n-3 polyunsaturated fatty acid levels found in spawning sardines compared to spawning anchovies indicate a poorer reproductive health status of sardine. By comparing the current total lipid content values with those recorded in other Mediterranean and North Atlantic areas, and others from more than 10 years ago, in the adjacent area of the Gulf of Lion, our study reveals the persistent poor condition of sardine and anchovy in the northern Catalan Sea. Furthermore, the low levels of diatom fatty acid markers observed throughout the spawning and non-spawning seasons in both sardine and anchovy indicate a diet poor in diatoms. Moreover, the results indicate that it is very unlikely that parasitism is a significant factor in the decline in condition of sardine and anchovy in the northern Catalan Sea. In fact, the results, which we believe provide useful insights for the management of small pelagic fisheries in the Mediterranean, suggest that the current poor condition of sardine and anchovy in the northern Catalan Sea has probably been exacerbated by a decrease in plankton productivity and/or a shift in the taxonomic composition of phytoplankton communities, adding to the ongoing effects of overfishing.

## Teaser

The evaluation of fatty acids and parasitism at different reproductive and feeding stages of sardine and anchovy in the northern Catalan coast reveals the current poor condition of these populations linked to potential changes in planktonic productivity in the context of changing environmental conditions.

## Introduction

Substantial declines in the stock size, mean body size and/or condition of European sardine (*Sardina pilchardus*) and European anchovy (*Engraulis encrasicolus*) have been observed in the north-western Mediterranean Sea since 2009 (Van Beveren *et al.*, 2014; Brosset *et al.*, 2015, 2016a, 2017; Ferrer-Maza *et al.*, 2016; Albo-Puigserver *et al.*, 2017, 2019; Saraux *et al.*, 2019), resulting in profound changes in the structure of the stocks and a major decline in the landings and fishing activity (Brosset *et al.*, 2017; Coll and Bellido, 2019; Saraux *et al.*, 2019). Similar negative trends in the body condition of sardine and anchovy have been documented in other northern areas of the Mediterranean Sea (Brosset *et al.*, 2017) and for sardine in the Bay of Biscay in the North Atlantic (Veron *et al.*, 2020). The current status of sardine and anchovy stocks is worrying as these small pelagic species are not only important to fisheries but they are also important from an ecological point of view, as they have a central place in the food web as forage species (Saraux *et al.*, 2019; Albo-Puigserver *et al.*, 2019). Forage fish play a fundamental role in marine trophodynamics because they uptake the energy available from low-level plankton and provide higher-order predators, including marine mammals, seabirds, large piscivorous fish and humans, with a highly nutritious and energetic food source (Cury *et al.*, 2000). Hence, changes in body condition in these small pelagic fish can have important implications for the whole ecosystem structure (Pethybridge *et al.*, 2014; Albo-Puigserver *et al.*, 2017, 2019; Saraux *et al.*, 2019).

Overfishing, climate change, diseases, predation by large fish such as tuna and competition between pelagic organisms for the zooplankton they feed on have all been suggested as factors to explain the decline in abundance and mean weight of sardine and anchovy populations in the Gulf of Lion. It seems, however, that the combined effects of poor condition, slower growth and the disappearance of older and larger individuals mediated by potential changes in food availability have been the major causes (Saraux *et al.*, 2014; Van Beveren *et al.*, 2014; Saraux *et al.*, 2019). In the NW Mediterranean, anchovy feed on zooplankton (particularly large copepods) whereas sardine feed on both zooplankton (mainly large copepods) and phytoplankton (mainly diatoms) (Plounevez and Champalbert, 2000; Costalago and Palomera, 2014; Le Bourg *et al.*, 2015). However, recent studies have suggested a shift in the diet of sardines in the Gulf of Lion from larger mesozooplankton (with a high proportion of cladocerans) before 2008 to smaller prey (copepods, suspected to be less nutritious) in the early 2010s (Zarubin *et al.*, 2014; Le Bourg *et al.*, 2015; Brosset *et al.*, 2016b). A combination of pollution and sea warming may have resulted in a long-lasting domination of smaller, lower-energy plankton in this region, which could be extremely detrimental to sardine populations (Queiros *et al.*, 2019). Overall, plankton composition, concentration and size seem to play a key role in determining the condition of small pelagic fish as other studies have shown: anchovy in the Strait of Sicily (Basilone *et al.*, 2004, 2006) and in the Adriatic (Zorica *et al.*, 2013), sprat in the Black Sea (Shulman *et al.*, 2005) and sardine in the Bay of Biscay (Veron *et al.*, 2020). In the Bay of Biscay, the decline in body condition in sardine since the late 2000s had no apparent link with fishing pressure but instead was linked to trophic responses involving a potential shift in the timing of the secondary production and/or the quality of the food (Veron *et al.*, 2020).

Assessing fatty acid composition in forage fish is seen as an ideal way to understand variability in their population dynamics (Shulman *et al.*, 2005; Litzow *et al.*, 2006; Lloret *et al.*, 2014; Pethybridge *et al.*, 2014; Keinänen *et al.*, 2017). In addition, fatty acid composition can be used to monitor energy availability and energy transfer in a food web because it is known to reflect the fatty acid content of the fish diet and, ultimately, of local phytoplankton (St John and Lund, 1996; Litzow *et al.*, 2006) and to determine the flow-on effects of these observed changes to their predators because lipid content in forage fish is likely to have a strong influence on the production of higher-level predators (Lloret *et al.*, 2014; Pethybridge *et al.*, 2014; Keinänen *et al.*, 2017).

Fatty acids are relevant from a nutritional point of view because they serve as substrates for a number of important metabolic energy and maintenance processes that underlie essential life history traits of fish, such as reproduction, growth and development. In particular, polyunsaturated fatty acids (PUFAs)—among which are the omega 3 fatty acids, such as eicosapentaenoic acid (EPA) and docosahexaenoic acid (DHA), and the omega 6 fatty acids, such as arachidonic acid (ARA)—are fundamental components of membranes, as they provide osmotic and electrolytic homeostasis and membrane permeability (Lloret *et al.*, 2014). In addition, n3-PUFAs have been identified as a major dietary factor in determining successful reproduction of fish, being crucial for the future requirements of the progeny (Tocher, 2003; Lloret *et al.*, 2014). They affect hatching success and viability of larvae because they are especially important in the development of larval activity and vision, as they accumulate in muscle, retinal rhodopsin and brain tissue of larvae and provide them with a better orientation during feeding (Tocher, 2003; Lloret *et al.*, 2014). EPA and ARA are precursors of prostaglandins, which have a role in final oocyte maturation and ovulation (Lloret *et al.*, 2014). In the case of sardine and anchovy, there is evidence of the importance of fatty acids in their reproduction success. For example, a significant variation in the EPA and ARA concentration of Iberian sardine oocytes was found to be caused by parental effects, with the amount, and particularly the composition, of the fat reserves that sardines are able to accumulate prior to the spawning season having a marked effect on the quality of the eggs produced during the spawning season (Garrido *et al.*, 2007). Hence, from an eco-physiological perspective, assessing PUFAs is one of the best ways to test the effects of lipid reserves on the reproductive success of small pelagic fish (Lloret *et al.*, 2014). However, the majority of marine fishes do not possess the ability to synthesize PUFAs themselves: in pelagic ecosystems, they are mostly produced only by phytoplankton and are transferred up the food webs; hence, they are considered to be essential fatty acids (EFAs; Dalsgaard *et al.*, 2003; Lloret *et al.*, 2014).

Furthermore, determining fatty acid profiles can help in monitoring ecosystem dynamics in the face of global climate change, reflecting baseline food web dependencies (Auel *et al.*, 2002; Dalsgaard *et al.*, 2003). In a changing ocean, studies of the fatty acid profiles of forage fish, complemented with other physiological measures such as oxidative stress balance, could help reveal shifts in primary productivity and consequently lead to a system-level understanding of marine trophodynamics (Litzow *et al.*, 2006; Pethybridge *et al.*, 2014; Queiros *et al.*, 2019).

Along with food availability and reproduction, parasitism has also been identified as a factor affecting the body condition of several fish species in the Mediterranean (e.g. Lloret *et al.*, 2012; Ferrer-Maza *et al.*, 2014, 2015, 2016; Serrat *et al.*, 2019). However, to our knowledge, few studies have looked into the effects of parasites on the lipid content of small pelagic fish in the Mediterranean, and those that exist have yielded conflicting results. For example, while Shchepkina (1985) and Ferrer-Maza *et al.* (2016) found an inverse relationship between parasitic infection rates and lipid concentration in muscle among anchovies in the Black Sea and the north-western Mediterranean Sea, a study by Van Beveren *et al.* (2016) found no evidence of a strong pathogenicity from parasites in sardine and anchovy in the Gulf of Lion.

In this context, this study analyses, from an ecological standpoint, the fatty acid composition of sardine and anchovy from the northern Catalan Coast (NW Mediterranean) in different reproductive and feeding stages, in order to assess the links between reproduction, feeding and lipid dynamics in both species. We also evaluate a number of fatty acid trophic markers that have been proposed as candidates for assessing changes in the condition of small pelagic fish related to changes in planktonic productivity. In addition, we compare the results provided in this paper in the northern Catalan Coast with results from other areas to shed light on the current situation in the northern Catalan Sea. Finally, the lipid dynamics are complemented with the analysis of an extensive parasitism data set in order to establish whether or not parasites are in some way responsible for the poor status of these small pelagic species in the study area.

## Methods

### Sampling of individuals

**Figure 1:**
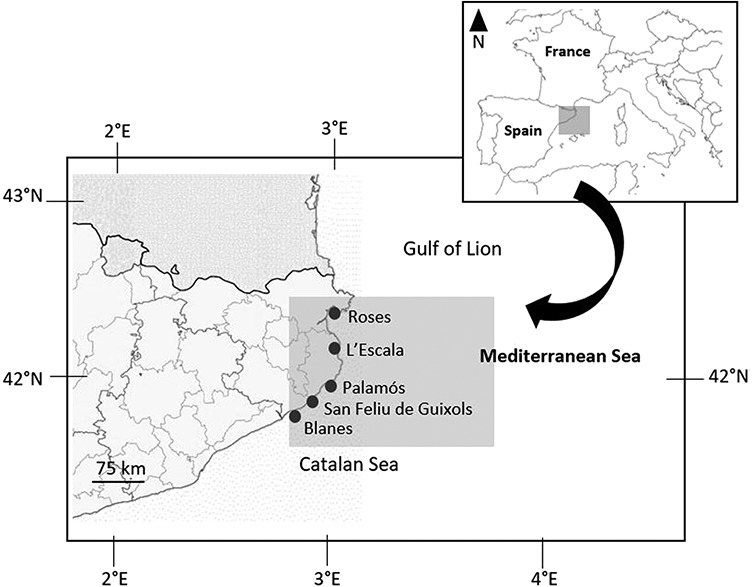
Map of the fishing ports in the northern Catalan Sea (NW Mediterranean) where samples of sardines and anchovies were taken.

Samples of adult sardines and anchovies caught by purse seines were taken at the ports of Blanes, Roses, Sant Feliu de Guíxols, L’Escala and Palamós in the northern Catalan Sea (Fig. 1), during two different periods, corresponding to the two spawning seasons reported for each species: first, the spawning season of sardine in autumn/winter period from November 2018 to January 2019 (Palomera and Olivar, 1996; Ganias *et al.*, 2007; Hani *et al.*, 2016); and second, the spawning season of anchovy in the spring/summer period from April 2019 to June 2019 (Palomera, 1992). In order to verify reproductive status, half the specimens sampled were assessed for maturity stage via visual inspection of the gonads after dissection. Based on collected commercial data and maturity stage descriptions for anchovy and sardine (ICES, 2008), we concluded that our spawning individuals were in the categories ‘spawning capable’ or ‘spawning’ (i.e. in the early and active reproductive periods) whereas non-spawning individuals were in the categories ‘post-spawning’, ‘resting’ or ‘developing’. Henceforth, we shall refer to these as ‘spawning anchovy/sardine’ and ‘non-spawning anchovy/sardine’. Inspecting the gonads of the specimens allowed us to relate lipid dynamics to reproductive cycle more precisely, compared to inferring the reproductive stage of the individuals from the month of capture, as was the case in previous works (e.g. Pethybridge *et al.*, 2014).

Sampling was performed on several days each month. Fish catches were grouped in different sample units, which were classified as follows: 10 samples of spawning sardines, 15 samples of non-spawning sardines, 9 samples of spawning anchovies and 13 samples of non-spawning anchovies. Sample units consisted of between 15 and 90 anchovies (similar lengths, randomly selected from the catch) and between 15 and 40 sardines (similar lengths, randomly selected from the catch). Because the effect of sex and length on lipid content and fatty acid levels of sardine and anchovy was not significant (as reported in the Gulf of Lion by Pethybridge *et al.*, 2014), we grouped the specimens together. Individuals were headed and gutted within 24 h of being caught and the muscle samples were homogenized with a grinder and kept frozen at −80°C until analysis.

### Analysis of total lipid (fat) and fatty acids

The fat or lipid content and fatty acid composition were determined for the muscle of sardine and anchovy, where both species, and indeed most pelagic fishes, store most of the energy reserves (review by Lloret *et al.*, 2014)**.** The total lipid content (% wet weight) was determined with an automatic Soxhlet extractor (Gerhardt SOX-416 Macro) following ISO 1443:1973 for fat extraction. Ground samples were first hydrolysed with hydrochloric acid (100 ml water+50 ml hydrochloric acid for every 10 grams of sample) and the lipid fraction was extracted by repeated extraction (percolation) with a volume of 150 ml of petroleum ether per 10 grams of sample. This solvent flowed for several cycles through the sample into a glass vitrified capsule (thimble) by distillation. The lipid content in the samples was then calculated by differences in weight.

Fatty acid methyl esters (FAMEs) were analysed by gas chromatography coupled with a flame ionization detector following ISO 12966-4:2015. First, 30 g of ground sample was extracted with 50 ml of petroleum ether. The extract was then evaporated by means of a Buchi rotary evaporator R-210. FAMEs were prepared by transesterification of the lipid extract, according to ISO 12966-2. FAMEs were analysed using an Agilent 7693A gas chromatograph coupled to a FID (Agilent Technologies, US). The injection volume of samples and standards was 1 μl and the column used was a high-polarity capillary column, BPX 70 (70% cyanopropyl/polysilphenylene-siloxane column, 30 m × 0.25 mm; 0.25 μm film thickness). Initial temperature was 90°C for 1 min, followed by a ramp of 4°C/minute up to 206°C and then another ramp of 20°C/minute up to 246°C at which point the temperature was held for 5 min. Detector and injector temperatures were set at 280°C and 260°C, respectively. The whole process lasted 37 min, with an air flow of 400 ml/min, an H_2_ flow of 30 ml/min and a helium flow of 25 ml/min. Chromatographic peaks were integrated and identified using standard samples (Supelco 37 Component FAME Mix, Sigma Aldrich). The content of each fatty acid in lipids was expressed as a percentage of the total content of all fatty acids. A total of 24 fatty acids were identified in the total lipid fraction from both species. However, some were detected at such low levels that a cut off point for quantification was set at 0.1% for both fish species. This resulted in the quantification of 16 fatty acids.

### Indices of trophic relationships

In order to assess trophic relationships, we computed the following ratios (Auel *et al.*, 2002; Dalsgaard *et al.*, 2003): palmitoleic acid/palmitic acid (16:1 n-7/16:0; or PO/P) and EPA/DHA (20:5 n-3/22:6 n-3). High values of these ratios indicate a diatom-based diet, whereas low values indicate a dinoflagellate-based diet. This is because among the specific lipid components suggested as suitable for use as trophic biomarkers in the pelagic marine environment (Dalsgaard *et al.*, 2003), diatoms contain high levels of PO, 16:1 n-7 and EPA, 20:5 n-3, whereas dinoflagellates usually contain elevated concentrations of stearidonic acid (18:4 n-3 or SDA) and DHA. Moreover, high EPA/DHA ratios indicate a diet that is predominantly carnivorous (zooplanktivorous), whereas low EPA/DHA ratios indicate a more herbivorous (phytoplanktivorous) diet (Dalsgaard *et al.*, 2003). High EPA/DHA ratios may also be indicative of an important influence of the primary production of cold-diatoms, since cold-water diatoms accumulate especially high amounts of EPA (Falk-Petersen *et al.*, 1998; Scott *et al.*, 1999).

### Evaluation of parasitism

To evaluate parasitism, we used the data provided by the Catalan Health Agency gathered from a 6-year programme (2002–2007) that monitored parasites in exploited fish species landed in Catalan ports (Servei de Veterinària de Salut Pública, 2007). Samples were collected randomly on a monthly basis by the agency’s veterinary inspectors at seven of the main fishing ports on the northern Catalan coast. The specimens were caught in the same areas (although in different years) where the specimens used to evaluate fatty acids were caught. In total, 1269 sardines (measuring between 11 and 31 cm) and 773 anchovies (measuring between 7 and 23 cm) were analysed for the presence of macroparasites. Immediately after landing, the inspectors recorded the total body length of each specimen and examined them for macroparasites in the gills, skin, fins and intestines using a binocular microscope in facilities at each port. When found, parasites were preserved in a lactophenol solution composed of 1:2:1 lactic acid, glycerol and water. The preserved parasites were then sent to the laboratories of the Catalan Centre of Microbiology where they were identified to the lowest possible taxa possible. 23% of sardine parasites and 16% of the anchovy parasites could be not identified. The prevalence of parasites was calculated as the proportion of fish infected with parasites, whereas the mean intensity of parasitism was calculated as the average number of parasites found in the infected hosts.

### Statistical tests

For each fish species, a multivariate ANOVA, considering the spawning and non-spawning period as a factor, was used to examine the existence of significant differences in fatty acid composition and fat content. Normality and homogeneity were verified using Kolmogorov–Smirnov and Levene tests. A *post hoc* Tukey's Honestly Significant Difference (HSD) was used to identify statistical differences between means. Fatty acid data were Hellinger transformed prior to performing a principal component analysis (PCA) analysis. The PCA was carried out to examine the relationships between fatty acid profiles, fatty acid ratios (PO/P and EPA/DHA) and fat content. Furthermore, for each species, the difference in the prevalence of parasites between spawning and non-spawning individuals was tested using a chi-square 2 × 2 contingency table. In all cases, the statistical significance was predetermined at *P* < 0.05. All analyses were performed using JMP13 software (SAS Institute, Cary, North Carolina, USA).

## Results

### Total lipid content (fat) and fatty acid profiles

The values for total lipid content (% wet weight) in the muscle tissue of sardine and anchovy are shown in Table 1. Mean lipid content was significantly lower in muscle from spawning sardine compared to non-spawning sardine (1.78% vs. 5.86%; *P* < 0.001). In contrast, lipid content was significantly higher in muscle from spawning anchovy compared to non-spawning anchovy (2.46% vs. 0.89%; *P* = 0.003).

**Table 1:** Total fat (% wet weight) and fatty acid profiles (% of total fatty acids) in the muscle of spawning and non-spawning sardine and anchovy^1^

	Sardine			Anchovy		
	Non-spawning	Spawning	*P*	Non-spawning	Spawning	*P*
Total fat	5.86 ± 2.14	1.78 ± 1.05	<0.001	0.89 ± 0.63	2.46 ± 1.49	0.003
C14:0	7.41 ± 0.90	8.07 ± 1.00	ns	6.90 ± 2.25	6.23 ± 1.74	ns
C16:0 (P)	23.75 ± 1.63	29.49 ± 7.35	0.007	28.89 ± 6.41	21.39 ± 3.50	0.005
C17:0	0.91 ± 0.16	1.44 ± 0.34	<0.001	1.37 ± 0.32	0.66 ± 0.44	<0.001
C18:0	4.45 ± 0.63	6.59 ± 1.55	<0.001	5.68 ± 1.13	2.93 ± 1.42	<0.001
C20:0	0.32 ± 0.12	0.43 ± 0.27	ns	0.24 ± 0.31	2.14 ± 1.22	<0.001
C22:0	0.15 ± 0.09	0.16 ± 0.10	ns	0.08 ± 0.10	0.04 ± 0.09	ns
C24:0	0.45 ± 0.45	0.15 ± 0.10	0.050	0.34 ± 0.68	0.12 ± 0.33	ns
Total SFA	37.49 ± 2.03	46.33 ± 8.76	0.001	43.50 ± 8.50	33.52 ± 5.68	0.006
C16:1 n-7 (PO)	5.59 ± 1.10	4.99 ± 1.24	ns	4.30 ± 1.33	3.84 ± 2.11	ns
C18:1 n-9	14.11 ± 1.37	12.66 ± 1.65	0.026	11.92 ± 1.73	9.69 ± 2.06	0.012
C20:1 n-9	3.57 ± 1.19	2.45 ± 1.97	ns	0.82 ± 0.95	1.52 ± 1.05	ns
C22:1 n-9	0.22 ± 0.21	0.23 ± 0.21	ns	0.10 ± 0.17	0.53 ± 0.22	<0.001
Total MUFA	23.61 ± 2.23	20.33 ± 4.48	0.023	17.15 ± 2.87	15.59 ± 4.63	ns
C18:3 n-3	1.95 ± 0.64	1.40 ± 0.16	0.016	1.07 ± 0.80	1.37 ± 0.86	ns
C20:5 n-3 (EPA)	10.91 ± 0.99	7.88 ± 1.42	<0.001	9.02 ± 1.58	14.13 ± 2.61	<0.001
C22:6 n-3 (DHA)	22.88 ± 2.38	20.86 ± 4.17	ns	25.24 ± 6.75	32.02 ± 7.46	0.038
Total n-3 PUFA	35.75 ± 2.70	30.13 ± 5.42	0.002	35.31 ± 8.01	47.53 ± 8.64	0.003
C18:2 n-6	2.26 ± 0.26	1.96 ± 0.12	0.003	1.92 ± 0.50	2.43 ± 0.33	0.014
C20:4 n-6	0.89 ± 0.21	1.22 ± 0.77	ns	2.12 ± 1.21	0.97 ± 0.15	0.010
Total n-6 PUFA	3.13 ± 0.18	3.19 ± 0.75	ns	4.03 ± 1.36	3.37 ± 0.46	ns
Total PUFA	38.88 ± 2.75	33.32 ± 5.82	0.004	39.34 ± 8.59	50.90 ± 8.89	0.006
PO/P	0.24 ± 0.05	0.18 ± 0.07	0.027	0.15 ± 0.05	0.18 ± 0.09	ns
EPA/DHA	0.48 ± 0.07	0.38 ± 0.04	<0.001	0.38 ± 0.09	0.45 ± 0.11	ns

^1^Pairs of means corresponding to spawning and non-spawning fish were compared; ns = not significant. Values in this table correspond to means ± standard deviation

The fatty acid compositions of the total lipid fraction (from the muscle in all cases) of both sardines and anchovies are presented in Table 1.

Saturated fatty acids (SFAs): between 37.49% and 46.33% of the total fatty acids in sardine and between 33.52% and 43.50% in anchovy were SFAs. The most abundant SFA in sardine and anchovy (spawning and non-spawning) was C16:0. Significant differences in the proportion of certain fatty acids between spawning and non-spawning fish were observed. The proportion of C16:0, C17:0 and C18:0—as well as total SFAs—was significantly higher in spawning sardine than in non-spawning sardine, while the reverse was true for C24:0, which was significantly higher in non-spawning sardine. In the case of anchovy, the proportion of C16:0, C17:0 and C18:0—as well as total SFAs—was significantly lower in spawning anchovy than in non-spawning anchovy, while the reverse was true for, in this case, C20:0, which was significantly higher in non-spawning anchovy (Table 1).

Monounsaturated fatty acids (MUFAs): between 20.33% and 23.61% of total fatty acids in sardine muscle and between 15.59% and 17.15% of total fatty acids in anchovy muscle were MUFAs. The most abundant MUFA in sardine and anchovy (spawning and non-spawning) was C18:1n-9. The proportion of C18:1n-9 and total MUFA was significantly lower in spawning sardines than in non-spawning sardines. In the case of anchovy, the proportion of C18:1n-9 was also significantly lower in spawning anchovy than in non-spawning anchovy, but the reverse was true for the proportion of C22:1n-9, which was significantly higher in non-spawning anchovy (Table 1).

PUFAs: between 33.32% and 38.88% of the total fatty acids in sardine muscle and between 39.34% and 50.90% in anchovy muscle were PUFAs, most of which were n-3 PUFAs (which comprised between 30.13% and 35.75% of total fatty acids in sardine and between 35.31% and 47.53% in anchovy). The main differences between the proportion of PUFAs in spawning and non-spawning individuals of both species involve n-3 PUFAs. Among the PUFAs, C22:6 n-3 (DHA) was present in the highest proportion in both species and in both spawning and non-spawning individuals. Significantly lower proportions of total PUFA, C20:5 n-3 (EPA), C18:3 n-3, C18:2 n-6 and n-3 PUFA were found in spawning sardines compared to non-spawning sardines; whereas, significantly higher proportions of C18:2 n-6, EPA, DHA and n-3 PUFA were found in spawning anchovy than in non-spawning anchovy. Only the proportion of C20:4 n-6 was found to be significantly lower in spawning anchovy than in non-spawning anchovy (Table 1).

### Principal component analysis

**Figure 2:**
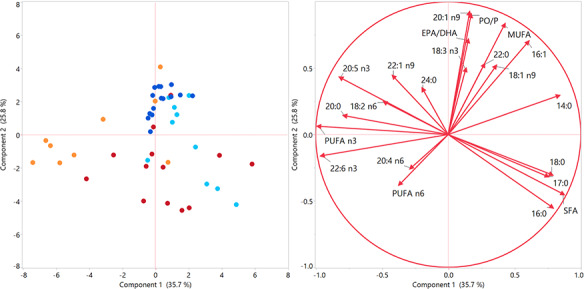
Plots of scores (non-spawning sardine = dark blue; spawning sardine = light blue; non-spawning anchovy = red; spawning anchovy = orange) and loadings of the PCA of fish species fatty acid composition.

A PCA was performed to examine the variation in fatty acid composition between the two fish species and period of spawning and to identify the fatty acids most responsible for this variation. The first two components of the PCA explained 61.5% of the variance. As shown in Figure 2, Component 1 influences the majority of SFAs and n-3 PUFAs. Component 1 positively influences C16:0, C17:0, C18:0 and total SFA and the ratios between C16:1/C16:0 (PO/P) and EPA/DHA, which are localized together and in opposite coordinates to C24:0, C20:5 n3, C18:2 n-6 and C22:1 n-9. In addition, C22:6 n-3, total n-3 PUFAs and C20:0 are grouped together and negatively influenced by Component 1. Meanwhile, Component 2 positively influences most of the MUFAs. Linolenic acid (C18:3 n-3) and C22:0 are also positively influenced by Component 2. Conversely, n-6 PUFA and C20:4 n-6 are negatively influenced by Component 2.

In general, the two PCA components allow the variability of fish species to be explained by the period of spawning. Component 2 is associated with the PO/P and EPA/DHA ratios and mainly explains the variability in the fatty acid profile of sardine due to the spawning period. On the other hand, it seems possible to separate spawning anchovies from non-spawning anchovies by the increase in EPA and DHA (as well as total n-3 PUFAs) and C20:0 and the decrease in the proportion of SFAs with a chain length of up to 18 carbons. Non-spawning sardines are also separated from non-spawning anchovies mainly due to the n-6 and n-9 fatty acid series (Component 2). Similarly, SFAs and n-3 PUFAs help to differentiate between spawning sardines and spawning anchovies (Component 1).

### Parasitism

All the parasites identified in sardines and anchovies were nematode larvae. The results of the chi-square tests for each fish species showed that the differences in the prevalence of parasites between spawning and non-spawning sardines and anchovies were insignificant. Therefore, the prevalence by species is presented for all individuals (spawning and non-spawning) taken together. Of all the dissected sardine specimens, 7.88% were infected with at least one nematode, with an intensity that ranged between one and three parasites (mean intensity = 1.15). *Hysterothylacium* sp. was the most frequent parasite, comprising 75.00% of the total nematodes identified, followed by *Anisakis* sp. (22.92% of the total). Of all the dissected anchovy specimens, 12.16% were infected with at least one nematode, with an intensity that ranged between one and four parasites (mean intensity = 1.10). Again, *Hysterothylacium* sp. was the most frequent parasite, comprising 70.40% of the total nematodes identified, followed by *Anisakis* sp. (28.61% of the total).

## Discussion

Our results provide new insights into lipid changes in sardine and anchovy that will contribute to our understanding of the physiology and ecology of these small pelagic species in the Mediterranean Sea in the face of changing environmental conditions.

### Seasonal variation in total lipid content and fatty acid profile in relation to reproduction and feeding cycles

First, this study demonstrates seasonal variations in total lipid content between spawning and non-spawning sardine and anchovy in the northern Catalan Sea, and these variations are linked to the different reproduction and feeding strategies of the two species. For sardine, our study found the lowest total lipid content values during the spawning season, i.e. in the autumn–winter period of low food (plankton) availability; for anchovy, the highest values were found during the spawning season, i.e. in the spring–summer period of high food (plankton) availability. Similar patterns of seasonal variability in total lipid content have already been reported in other studies (Ganias *et al.*, 2007; Pethybridge *et al.*, 2014; Ferrer-Maza *et al.*, 2016; Albo-Puigserver *et al.*, 2019) and are in consonance with the breeding strategy of each species: sardine has been described mainly as a capital breeder, relying on energy stores accumulated prior to reproduction, whereas anchovy has been described mainly as an income breeder, relying on an abundant food source during their spawning phase (García and Palomera, 1996; Somarakis, 2004; McBride *et al.*, 2015; Brosset *et al.*, 2016b).

Second, our study has been able to link the fatty acid composition of sardine and anchovy in the northern Catalan Sea with the reproductive and feeding cycle of these species. In the case of sardine, and in line with the total lipid content data, levels of total MUFAs and total PUFAs were highest during the non-spawning season during which feeding intensity is high, in consonance with capital breeding strategy. In the case of anchovy, and also in line with the total lipid content data, the total PUFAs were significantly higher during the spawning season, during which the phytoplankton are in maximum supply, in consonance with its income breeding strategy. However, while the differences in MUFAs between spawning and non-spawning anchovies were not significant, the total SFA values in both species displayed the opposite pattern to that of total lipid with significantly higher values in spawning sardine and in non-spawning anchovy. There are, therefore, complex trade-offs between reproduction, growth and basal energy that cannot be fully explained in our study.

The reproductive and feeding cycles of these two fish species explain the variability in the fatty acid composition that can be differentiated by means of a PCA. Similar findings have been reported for the fatty acid composition of sprat, sardine and anchovy collected in Gulf of Lion (Pethybridge*et al.*, 2014). These authors reported that C14:0, C16:0, C16:1 n-7, C18:1 n-9, EPA and DHA are crucial for explaining the variability in the fatty acid composition of these fish species, which is very consistent with our findings. It is also worth noting that the PO/P and EPA/DHA ratios together with the proportions of C18:1 n-9, and, to a lesser extent, EPA and C14:0, can help to explain feeding habits. It appears, therefore, that the sardine’s diet during spawning is more carnivorous (zooplanktivorous) and less herbivorous (phytoplanktivorous) than it is during non-spawning. However, these indices lack significance in the case of anchovy, which suggests that it has different feeding habits compared to sardine, supporting the hypothesis that sardine and anchovy probably do not compete strongly for food resources (Chouvelon *et al.*, 2015). As shown in the PCA, higher percentages in DHA seem to discriminate anchovies during spawning when their diet may be richer in dinoflagellates and, in general, phytoplankton.

### The relevance of PUFAs

The variation in total PUFA levels found in sardine and anchovy in the northern Catalan Sea was mostly due to variations in the levels of highly unsaturated fatty acids, namely the n-3 fatty PUFAs (EPA and DHA). Although the relative proportion of n-3 PUFAs in the fatty acids of non-spawning sardine and non-spawning anchovy was quite similar (about 35%), the relative proportion in spawning sardine was much lower (about 30%) than in spawning anchovy (47%). If we consider the significant relationships found between n-3 PUFAs in female muscle and oocytes of sardine in the North Atlantic, and the relationship between female diet—in particular, plankton availability immediately before and during the spawning season—and the quality of offspring produced by sardine (Garrido *et al.*, 2007), then we can surmise that the relatively low proportion of n-3 PUFAs in spawning sardines in the northern Catalan Sea indicates a poorer reproductive status of this species than that of anchovy. n-3 PUFAs have been identified as a major dietary factor determining successful reproduction in fish, as they are crucial for the future requirements of the eggs and larvae (Bruce *et al.*, 1999; Tocher, 2003; Lloret *et al.*, 2014). It must be also taken into account that sardines have a lower degree of trophic plasticity than anchovies, both in terms of feeding areas and in the size of the zooplanktonic prey consumed (Chouvelon *et al.*, 2015) and that Van Beveren *et al.* (2016) revealed elevated quantities of macrophage aggregates in sardines in the Gulf of Lion indicating stress on the fish that might potentially be related to starvation. In the following section, we address the issue of the challenging food supply over time in more detail.

### What do fatty acids tell us regarding the current status of sardine and anchovy stocks under challenging environmental conditions?

In order to understand the challenges facing small pelagic fish in the Mediterranean, and particularly that of sardine, we shall now discuss how the fatty acid profiles, and the ratios computed, help to explain the potential causes behind the current status of the stocks. In our study, low diatom markers were present throughout the spawning and non-spawning seasons of sardine and anchovy. The low (< 0.50) ratios of PO/P and EPA/DHA for both sardine and anchovy during the non-spawning periods support the hypothesis of a diet for both species that is not predominantly based on diatoms. This is in contrast to the situation 10 years ago (2010 and 2011) in the adjacent waters of the Gulf of Lion, where for all seasons, these ratios indicated a predominantly diatom-based diet for both species (Pethybridge *et al.*, 2014). In fact, studies on stomach analyses of sardine in the Gulf of Lion at that time (2011–2012) showed a higher proportion of diatoms in the diet compared to dinoflagellates, a situation that was more accentuated during summer when diatom abundance was usually high after the spring bloom (Leblanc *et al.*, 2003; Le bourg *et al.*, 2015).

Furthermore, the relatively low PO/P and EPA/DHA ratios of non-spawning sardine in the northern Catalan Sea compared to the ratios observed in other Mediterranean and North Atlantic areas indicate that, in the Catalan Sea, the proportion of diatoms in the diet of sardine is lower than in other areas (Table 2). Furthermore, the comparatively lower EPA/DHA values and high C16:0 values of non-spawning sardines in the northern Catalan Sea suggest that low-energy phytoplankton is proportionally more important than high-energy zooplankton in the sardine’s diet in the study area compared to other areas. This pattern does not occur in anchovy (Table 3), for which the ratios of PO/P, EPA/DHA and the levels C16:0 in non-spawning individuals from the Catalan Sea are similar to other Mediterranean areas, except in the Black Sea, where higher PO/P and EPA/DHA values and lower C16:0 values are found (Table 3). Notwithstanding these results, the comparison of fatty acid profiles between areas must be taken with caution because values compared are expressed in % of total fatty acid mass, and it would be much better to compare data on absolute fatty acid content (% body mass) (Litzow *et al.*, 2006).

**Table 2:** Total fat (% wet weight) and fatty acid profiles (% of total fatty acids) during non-spawning periods for *Sardina pilchardus* in the Eastern Algarve waters of the Atlantic Ocean (Bandarra *et al.*, 2018), the Mediterranean waters of the Adriatic Sea (De Leonardis and Macciola, 2004), the Gulf of Lion (Pethybridge *et al.*, 2014) and the northern Catalan Sea (our study)

	Eastern Algarve	Adriatic Sea	Gulf of Lion	This study (N Catalan Sea)
Marine areas	October 2016	January–March and August–October 2002	July 2010	November–April 2019–2020
Total fat	14.0	(−)	18.21	5.86
C16:0 (P)	19.6	22.3	(−)	23.75
C16:1 n-7 (PO)	6.6	9.2	(−)	5.59
PO/P	0.34	0.41	(−)	0.24
C20:5 n-3 (EPA)	13.6	6.5	(−)	10.91
C22:6 n-3 (DHA)	14.8	11.3	(−)	22.88
EPA/DHA	0.92	0.57	(−)	0.48

(−) means no data available.

**Table 3:** Total fat (% wet weight) and fatty acid profiles (% of total fatty acids) during non-spawning periods for *Engraulis encrasicolus* in the Mediterranean Sea (Eastern Mediterranean and Black Sea) (Oksuz *et al.*, 2009); Tyrrhenian Sea, Adriatic Sea and Ionian Sea (Roncarati *et al.*, 2012); Gulf of Lion (Pethybridge *et al.*, 2014); and northern Catalan Sea (our study)

Marine Areas	Eastern Medit.	Black Sea	Tyrrhenian Sea	Adriatic Sea	Ionian Sea	Gulf of Lion	This study (N Catalan Sea)
	December 2007	January 2009	May 2007–2008 and November 2007	May 2007–2008 and November 2007	May 2007–2008 and November 2007	March 2011	December–June 2019–2020
Total fat	(−)	8.85	2.27	1.81	1.91	8.19	0.89
C16:0 (P)	22.13	17.35	23.66	23.27	21.94	(−)	28.89
C16:1 n-7 (PO)	2.61	7.93	2.95	3.17	3.71	(−)	4.30
PO/P	0.12	0.46	0.12	0.14	0.17	(−)	0.15
C20:5 n-3 (EPA)	5.65	10.92	6.13	6.25	6.49	(−)	9.02
C22:6 n-3 (DHA)	33.4	15.29	28.06	27.16	25.28	(−)	25.24
EPA/DHA	0.17	0.71	0.22	0.23	0.26	(−)	0.36

(−) means no data available.

Although our results show relatively similar or higher values of EFAs in non-spawning sardine and anchovy compared to other Mediterranean and Atlantic areas (Tables 2 and 3), when we compare the level of total fat content of non-spawning sardine and anchovy in our study area with that of the Gulf of Lion 10 years ago (Pethybridge *et al.*, 2014), we can conclude that the total fat content in the muscle of both species has declined (Tables 2 and 3). This may be related to a decrease in primary production, since a recent study in a coastal area of the northern Catalan Sea showed that most of the phytoplankton groups there presented a decreasing trend in abundance, which could be associated with a reduction in nutrient availability (Nunes *et al.*, 2018). Furthermore, the total lipid content of non-spawning sardines and anchovies in the northern Catalan Sea is also much lower than in the other areas (Tables 2 and 3) suggesting that sardine may even be forced to rely on direct food intake to acquire enough energy during the spawning period.

Taking all these results together, it appears that a decrease in plankton productivity and/or a shift in the taxonomic composition of phytoplankton communities may have occurred in the northern Catalan Coast in the last decade, although further investigations are needed to confirm this statement. By altering the pelagic environment, climate change may be altering the composition and distribution of plankton species, as well as their importance in the food web, with higher temperatures favouring the smallest components of the plankton, thus strengthening microbial loop activity (Thiébault *et al.*, 2016). As increasing temperature favours planktonic organisms of smaller size, climate change may particularly affect the condition of sardine, as recent results revealed that food size (without any modification of its energy content) is as important as food quantity for the body condition, growth and reserve lipids of sardine (Queiros *et al.*, 2019). Furthermore, fluctuations in the diatom/dinoflagellate ratio may have ecosystem-wide consequences for the transfer of energy and matter to higher trophic levels considering the importance of both groups in the trophic chain of many seas (Wasmund, 2017).

### Parasitism

Our results on parasitism showed that the metazoan parasite fauna of sardine and anchovy in the northern Catalan Sea is dominated by nematode larvae. However, considering the low prevalence and low intensity of parasites in sardine and anchovy in the study area, our results are in line with Van Beveren *et al.* (2016) and Saraux *et al.* (2019) both of which concluded that it is very unlikely that parasites, or any other pathogenic agent, are root causes of the drastic population modifications observed in sardine and anchovy in the NW Mediterranean. Only the study by Ferrer-Maza *et al.* (2016) showed that certain species of parasites had a negative effect on female egg production and lipid content in smaller anchovies. However, there is a wide range of research available concerning the nematodes that infect anchovy and sardine in the Mediterranean (e.g. Rello *et al.*, 2008, 2009; Gutiérrez-Galindo *et al.*, 2010; Cavallero *et al.*, 2015; Zorica *et al.*, 2016) and, in general, such studies have reported a relatively low prevalence of nematodes in these two species, ranging from 0 to 25%, with low values for overall prevalence when data from different studies were pooled (<3% in the case of Anisakids in the meta-analysis conducted by Colombo *et al.*, 2016). Although it is true that prevalence may change depending on season, parasite species and area (Mladineo *et al.*, 2012; Zorica *et al.*, 2016), such low values support the idea that parasites are probably not responsible for the poor status of sardine and anchovy stocks in the Mediterranean.

Our results contribute to the literature on parasitic infestation of sardine and anchovy in the northern Catalan Sea because, for the first time, we used extensive data from a monitoring programme covering many ports and years and seasons. Although other metazoan parasites (e.g. Digenea and Cestoda, which are found in particular organs, such as pyloric caeca, and the stomach, and tissues such as muscle) are difficult to evaluate in the port and may have gone undetected, the evaluation carried out by the veterinarian inspectors involved in the monitoring programme in the northern Catalan Sea ports provides good estimates of parasitism in this region.

## Conclusion

The results from this study provide evidence that fatty acid levels are good indicators not only of the body condition at different reproductive and feeding stages of small pelagic fish but also of pelagic environmental conditions in the context of global change. The study contributes to our understanding of the relationships between condition, reproduction and feeding in sardines and anchovies through the analysis of variations in the profiles of fatty acids that are crucial in key life history traits of these forage species, particularly reproductive success. In our study, low diatom markers were observed throughout the spawning and non-spawning seasons of sardine and anchovy, suggesting a potential shift in the diets of sardine and anchovy in the area from diatoms to dinoflagellates. Overall, these results suggest that a decrease in plankton productivity and/or a shift in the taxonomic composition of phytoplankton communities may have occurred in the northern Catalan Sea in the past decade. Feeding conditions in spring and summer appear to be a key factor in determining not only total lipid content but also the levels of specific fatty acids of sardine and anchovy in the NW Mediterranean. According to our results, it would appear that, based on total lipid content and fatty acid distribution, sardine and, to a lesser extent, anchovy in the northern Catalan Sea are currently in poor condition and malnourished. In order to confirm this trend and to confirm any potential limitations in PUFA/EFA availability, further studies on the fatty acids of small pelagic fish in the area will be needed.

Furthermore, further studies on the planktonic composition and its evolution in the Mediterranean Sea are needed to improve our understanding of the impacts of changing food quantity and quality on the condition of these small pelagic fish, which may have consequences not only for fisheries but also for the whole trophic chain—considering their importance as forage species. Furthermore, bearing in mind that, in the Catalan Sea, which is part of GSA6, the sardine and anchovy stocks are considered to be overexploited according to STECF assessments (STECF, 2017), the poor condition status of these species adds to the worries posed by the impact of fishing activity. This is more evident for sardine, which according to assessments made by GFCM (2017), has seen a negative trend in landings and acoustic biomass estimates since 1994 in GSA 6.

Our results also support the idea that it is very unlikely that parasites are a root cause of the decline in the condition of sardine and anchovy in the NW Mediterranean, but any increase in parasitism could instead be the result of qualitative and/or quantitative modifications in planktonic production leading to fish in poorer condition, particularly sardines. Hence, further studies of parasitism in sardine and anchovy in the NW Mediterranean will probably not provide much useful information for stock conservation purposes. Likewise, the use of simple morphometric, physiological and bioenergetic condition indices—such as Le Cren’s K_n_, total lipid content or energy density, which have been widely used for small pelagic fish in the Mediterranean (Ferrer-Maza *et al.*, 2016; Albo-Puigserver *et al*., 2017; Brosset *et al.*, 2017)—are less likely to yield significant advances in relation to fish conservation because these indicators of fish condition are not directly linked (in the way that EFAs are) to the life history traits of fish (Lloret *et al.*, 2014). Therefore, from a perspective of fish and fisheries conservation, it is important that further work is carried out to assess the links between the fatty acid composition of sardines and anchovies—particularly the Omega-3 fatty acids—and their biological traits, such as growth, reproduction (e.g. quantity and quality of eggs and larvae) and natural mortality. In this way, the monitoring of EFAs in small pelagic fish could provide an indicator of the overall health of the individuals, which could then be used to better forecast the trends in abundance and biomass of the populations in the context of standard stock assessments. Furthermore, considering the importance of sardines and anchovies in the diet of predatory pelagic fish in the western Mediterranean Sea, such as swordfish, *Xiphias gladius*, and more particularly little tunny, *Euthynnus alletteratus*, and Atlantic bonito, *Sarda sarda* (Navarro *et al.*, 2017), the evaluation of EFAs in small pelagic fish could be used not only to refine management strategies that promote the conservation of stocks of these fish but also those of their predators, optimizing the economic benefits within an ecosystem-based approach to fisheries management (Pikitch *et al.*, 2004).

Although several condition indices have been used in a wide variety of contexts in conservation and environmental research involving different marine fish species, integrating these indices—particularly the physiological ones—into stock assessment and management is difficult and has been carried out in only a few cases (Young *et al.*, 2006; Lloret *et al.*, 2012). Our results support the idea that fatty acids can be used as indicators to gather valuable information on the reproductive biology and feeding ecology of small pelagic fish in a changing ocean environment. Because these variables can influence the abundance and distribution of fish, in practice they could provide useful insights for the management of small pelagic fisheries and could be used, for example, to help develop hypotheses for assessment, to plan fishing periods or area closures, to increase public awareness and to promote conservation (Young *et al.*, 2006; Lloret *et al.*, 2012). In particular, a better understanding is needed of the link between fatty acid composition in fish and the population parameters used in standard stock assessments, such as natural mortality, growth and fecundity, in order to improve accuracy in determining biological reference points. Second, there is a need to explore the use of fatty acids in small pelagic fish as an additional ecosystem indicator for fisheries management and ecosystem health as an alternative to other indicators proposed by various authors (e.g. Cury and Christensen, 2005) and policy documents (e.g. the Marine Strategy Framework Directive from the European Union).

## Funding

This study was supported by FLAG/GALP-Costa Brava projects Ref. ARP014/17/00007 and Ref. ARP014/17/00054 (financed by the European Maritime and Fisheries Fund—EMFF and the DG Fisheries of the Catalan Government).
